# Development and validation of a nomogram for predicting the risk of obstructive coronary artery disease in rheumatoid arthritis patients based on LDL-C, Th17 cells, and IL-17

**DOI:** 10.3389/fimmu.2024.1493182

**Published:** 2024-12-17

**Authors:** Xiaoyang Wang, Baochen Li, Ruipeng Wei, Bin Hu, Yuming Feng, Bin Yang, Shuling Rong, Bao Li

**Affiliations:** ^1^ Department of Cardiology, Second Hospital of Shanxi Medical University, Taiyuan, Shanxi, China; ^2^ Department of Rheumatology, Second Hospital of Shanxi Medical University, Taiyuan, China

**Keywords:** rheumatoid arthritis, obstructive coronary artery disease, nomogram, LDL-C, Th17 cells, IL-17

## Abstract

**Objective:**

This study aims to develop and validate a nomogram model for predicting the risk of obstructive coronary artery disease (CAD) in patients with rheumatoid arthritis (RA), incorporating low-density lipoprotein cholesterol (LDL-C), Th17 cells, and interleukin (IL)-17 levels. The proposed model seeks to enable personalized cardiovascular risk assessment for RA patients, thereby optimizing clinical management strategies.

**Methods:**

A total of 120 patients with rheumatoid arthritis (RA) who were treated at the Second Hospital of Shanxi Medical University between January 2019 and September 2023 were enrolled in this study. Based on coronary angiography results, patients were categorized into the RA-obstructive CAD group and the RA-non-obstructive CAD group. Additionally, 53 healthy controls (HC group) were included. Clinical characteristics, laboratory parameters, peripheral blood lymphocyte subsets, and cytokine levels were collected for analysis. Univariate logistic regression was used to identify risk factors associated with RA-obstructive CAD. These variables were further refined using a random forest model for optimal selection. Finally, multivariate logistic regression analysis was performed with the selected variables to develop a nomogram model, which was subsequently validated to assess its performance.

**Results:**

Compared with the RA-non-obstructive CAD group, the RA-obstructive CAD group demonstrated significantly elevated levels of immune cell subsets, such as Th17 cells, and cytokines, including IL-17, IL-2, and IL-4, along with a reduction in Treg cells. (2) In the training cohort, univariate and multivariate logistic regression analyses identified LDL-C (OR = 0.04, P < 0.001), Th17 cells (OR = 0.76, P = 0.005), and IL-17 (OR = 0.75, P = 0.001) as independent risk factors for obstructive CAD in RA patients. Subsequently, a predictive nomogram model for RA-obstructive CAD risk was developed based on these indicators, incorporating LDL-C, Th17 cells, and IL-17.

**Conclusion:**

This study developed a predictive nomogram for RA-obstructive CAD by combining traditional risk factors, such as LDL-C, with immune biomarkers Th17 and IL-17. The model demonstrated robust predictive accuracy, enabling more precise risk assessment of CAD in RA patients. It offers clinicians a valuable tool for advancing cardiovascular risk management in RA, underscoring its significant potential for clinical application.

## 1 Introduction

Rheumatoid arthritis (RA) is a prototypical immune-mediated autoimmune disease, primarily characterized by chronic synovitis and vasculitis, which can ultimately lead to joint dysfunction. Beyond local joint damage, RA induces systemic chronic inflammation, significantly increasing the risk of atherosclerosis and obstructive coronary artery disease (CAD) (1, 2). Compared to the general population, RA patients experience a 48% higher incidence and a 50% higher mortality rate from cardiovascular disease (CVD) (3, 4). Even after controlling for traditional cardiovascular risk factors such as smoking, hypertension, and hyperlipidemia, approximately 50% of RA patients still develop atherosclerosis (5–7). Therefore, the specific predictors of obstructive CAD risk in RA remain insufficiently defined.

Recent studies have elucidated the pivotal role of immune dysregulation and persistent inflammation in the pathogenesis of both RA and obstructive CAD (8, 9). In RA, an imbalance in peripheral Th17/Treg cell populations, characterized by an increase in pro-inflammatory Th17 cells and a reduction in Treg cell function, plays a critical role in the development of atherosclerosis (10). Th17 cells primarily modulate immune responses through the secretion of cytokines such as IL-17, IL-21, and IL-22, with IL-17 playing a central role in the inflammatory processes associated with RA and obstructive CAD (11–14). IL-17 not only activates synovial cells to secrete inflammatory mediators, exacerbating joint inflammation (15, 16), but also induces the production of pro-inflammatory factors by fibroblasts and endothelial cells, stimulates smooth muscle cell proliferation, and promotes arterial wall inflammation, thereby driving the progression of atherosclerosis (15). Treg cells, which are critical in maintaining immune tolerance and suppressing excessive inflammation, are often dysfunctional in RA patients, leading to immune imbalance and promoting the overactivation of Th17 cells, further exacerbating inflammatory responses (17). The reduction of Treg cells not only intensifies joint inflammation but also diminishes their protective role in endothelial cells, thereby increasing vascular inflammation (18). The interplay between excessive Th17 activation and impaired Treg function represents a key driver of vascular pathology in both RA and obstructive CAD, forming a complex immunological network that underpins their pathogenesis.

Despite the established association between immune dysregulation, inflammation, and increased cardiovascular risk in RA, these factors have not been adequately incorporated into most existing cardiovascular risk assessment tools. Current models, such as the Framingham Risk Score (FRS) and SCORE, fail to sufficiently account for the impact of inflammation and immune imbalance on atherosclerosis, often underestimating the CAD risk in RA patients (19, 20). Although the ERS-RA risk score proposed by Solomon et al. (21) combines traditional cardiovascular risk factors with RA-related markers (e.g., inflammation, disease duration, and corticosteroid use), its predictive performance does not surpass that of the FRS. Therefore, there is a pressing need for a dedicated obstructive CAD risk prediction model specifically tailored for RA patients.

This study aims to analyze the clinical characteristics and immunological differences in RA patients with obstructive CAD, integrating serum lipid profiles, immune cell populations, and cytokine levels to develop and validate a personalized cardiovascular risk prediction nomogram. This model is designed to enable clinicians to provide more precise cardiovascular risk assessments and tailored therapeutic strategies for RA patients. By facilitating early identification of high-risk individuals and implementing effective preventive measures, the model has the potential to reduce cardiovascular events, enhance treatment outcomes, and ultimately improve patient quality of life and survival. These findings hold significant clinical value for optimizing patient care.

## 2 Materials and methods

### 2.1 Clinical characteristics

This study included 120 patients diagnosed with RA who attended the Rheumatology Department of the Second Hospital of Shanxi Medical University between January 2019 and September 2023. Based on prior coronary angiography results, patients were divided into two groups: 60 RA patients with obstructive coronary artery disease (RA-obstructive CAD) and 60 RA patients without obstructive CAD (RA-non-obstructive CAD). No significant differences were observed between the two groups in terms of age, sex, or disease duration. Additionally, 53 healthy individuals undergoing routine health examinations at our hospital’s physical examination center were included as the healthy control group (HC group). All RA patients met the 2010 classification criteria for RA established by the American College of Rheumatology/European League Against Rheumatism (ACR/EULAR) (22).

Inclusion criteria for the RA-obstructive CAD group were as follows: patients who underwent coronary angiography due to chest discomfort, chest pain, or abnormal electrocardiogram findings, with a diagnosis of CAD based on the 2016 American Heart Association guidelines (23). Exclusion criteria included the presence of other autoimmune diseases, coronary branch lesions (e.g., lesions in the diagonal branch of the left anterior descending artery, obtuse marginal branch of the left circumflex artery, or posterior descending branch of the right coronary artery), left main coronary artery disease, a history of non-ST-elevation myocardial infarction (NSTEMI) or ST-elevation myocardial infarction (STEMI), other cardiovascular diseases (e.g., heart failure, congenital heart disease, or valvular heart disease), malignancies, infections, or severe dysfunction of other organs.

Inclusion criteria for the RA-non-obstructive CAD group were as follows: patients presenting with chest discomfort, chest pain, or abnormal electrocardiogram findings, with coronary angiography confirming the absence of significant stenosis or mild stenosis (<50%) consistent with the expert consensus of the European Association of Percutaneous Cardiovascular Interventions (24). Exclusion criteria mirrored those of the RA-obstructive CAD group, including other autoimmune diseases, coronary branch lesions (e.g., lesions in the diagonal branch of the left anterior descending artery, obtuse marginal branch of the left circumflex artery, or posterior descending branch of the right coronary artery), left main coronary artery disease, malignancies, infections, severe cardiovascular diseases, and significant dysfunction of other organs.

Exclusion criteria for the HC group included: autoimmune diseases; cardiovascular diseases (e.g., coronary artery disease, heart failure, hypertension, congenital heart disease); metabolic diseases; cancers; infections or patients undergoing antimicrobial therapy; and individuals with abnormal liver or kidney function.

This study was approved by the Medical Ethics Committee of the Second Hospital of Shanxi Medical University (approval number [2024]YX-292). Clinical and laboratory data were retrospectively collected from the clinical database. Clinical characteristics included age, sex, body mass index (BMI), traditional cardiovascular risk factors, clinical cardiology diagnoses, and medication usage. Laboratory tests included the Disease Activity Score 28 (DAS28), rheumatoid factor (RF), anti-citrullinated protein antibody (Anti-CCP), erythrocyte sedimentation rate (ESR), C-reactive protein (CRP), routine blood count, liver and kidney function tests, lipid profile, immunoglobulins, absolute counts and frequencies of peripheral blood lymphocyte subsets, and cytokine levels.

### 2.2 Detection of the absolute and relative counts of peripheral blood lymphocyte subsets by using flow cytometry

Whole blood was collected from all patients using heparinized tubes, and peripheral blood mononuclear cells (PBMCs) were isolated by density gradient centrifugation with Ficoll-Hypaque. Centrifugation conditions were set at room temperature for 20–30 minutes at 800–1,000 × g, and the resulting PBMCs were resuspended to an appropriate concentration. Lymphocyte subsets in peripheral blood were then sequentially stained with fluorescence-labeled monoclonal antibodies. Specifically, anti-CD3-FITC, anti-CD8-PE, anti-CD45-PercP, and anti-CD4-APC were used to stain T lymphocytes; anti-CD3-FITC, anti-CD16+CD56-PE, anti-CD45-PercP, and anti-CD19-APC were used for B lymphocytes and NK cells; anti-CD4-FITC and anti-IFN-γ-APC for Th1 cells; anti-CD4-FITC and anti-IL-4-PE for Th2 cells; anti-CD4-FITC and anti-IL-17A-PE for Th17 cells; and anti-CD4-FITC, anti-CD25-APC, and anti-FOXP3-PE for Treg cells. All fluorescence-labeled monoclonal antibodies used in this study were purchased from BD Biosciences (Franklin Lakes, NJ, USA) and the experiments were conducted according to the manufacturer’s instructions. Absolute and relative counts of peripheral blood lymphocyte subsets were determined using a FACSCalibur flow cytometer and BD Multitest software (BD Biosciences, Franklin Lakes, NJ, USA).

### 2.3 Cytokine levels assessed by bead-based multiplex immunoassay

Blood samples from all patients were centrifuged within 1 hour of collection to obtain serum, which was stored at -20°C until analysis, with a maximum storage time of 96 hours. Serum were separated under the following centrifugation conditions: room temperature, 15-20 minutes, 1,500-2,000 × g. Serum levels of seven cytokines—interleukin (IL)-2, IL-4, IL-6, IL-10, IL-17, tumor necrosis factor-α (TNF-α), and interferon-γ (IFN-γ)—were measured using a bead-based multiplex immunoassay. The Th1/Th2/Th17 cytokine detection kits were purchased from Cellgene Biotechnology Co., Ltd. (Jiangxi, China) and assays were conducted following the manufacturer’s protocol. Cytokine concentrations (pg/mL) were determined using the BioPlex 200 system, and data analysis was performed with BioPlex Manager software.

### 2.4 Statistical analysis

In this study, the normality and homogeneity of variance of the data were first assessed using the Kolmogorov-Smirnov test and Levene’s test. For data with a normal distribution, means ± standard deviations (SD) were used, and between-group comparisons were performed using independent sample t-tests. For data that were not normally distributed, medians (interquartile range, IQR) were used for description, and Kruskal-Wallis H tests were applied for between-group comparisons. Categorical data were expressed as frequencies, and between-group comparisons were performed using chi-square tests. To adjust for the effects of covariates, analysis of covariance (ANCOVA) was used to test the differences between groups. All statistical analyses were conducted using SRA 27.0 software (SRA Inc., Chicago, IL, USA).

The study population of 120 RA patients was randomly divided into a training group (96 patients) and a validation group (24 patients) in an 8:2 ratio using a random number table. First, univariate logistic regression was performed to identify risk factors associated with RA complicated by obstructive CAD, and statistically significant variables were incorporated into a random forest model. Variables were ranked according to their mean decrease in Gini (MDG) values from the random forest model, and variables were progressively selected for multivariate logistic regression analysis based on the lowest out-of-bag error estimate (OBB). A nomogram prediction model was constructed based on the identified risk factors. The discriminative ability of the model was evaluated using the area under the receiver operating characteristic curve (AUC). Calibration was verified using a calibration curve and the Hosmer-Lemeshow test, while clinical utility was assessed via decision curve analysis (DCA). All data analyses and visualizations were performed using R software (version 3.6.3, R Foundation for Statistical Computing, Vienna, Austria).

## 3 Results

### 3.1 Comparison of baseline characteristics, clinical features, and laboratory data among RA-obstructive CAD, RA-non-obstructive CAD, and HC groups

This study included a total of 120 patients (42 males, 78 females; mean age 66.37 ± 10.24 years) and 53 healthy controls (17 males, 36 females; mean age 48.19 ± 11.87 years). The baseline demographic information, clinical features, and laboratory data for all participants are summarized in **Table 1**. Among the RA-obstructive CAD and RA-non-obstructive CAD groups, some patients received medication, including nonsteroidal anti-inflammatory drugs (NSAIDs), conventional synthetic disease-modifying antirheumatic drugs (csDMARDs), biologic agents (bDMARDs), and corticosteroids (GC), with no significant differences between the two groups. Additionally, some RA-obstructive CAD patients were treated with statins, antiplatelet agents, β-blockers, renin-angiotensin-aldosterone system inhibitors, angiotensin-converting enzyme inhibitors, and other vasodilators.

**Table 1:** Clinical Characteristics of the RA-Obstructive CAD, RA-Non-Obstructive CAD, and HC Groups.

	RA-Obstructive CAD (n=60)	RA-Non-Obstructive CAD (n=60)	HC (n=53)	*P*
Demographics				
Age (Years)^a^	66.92 ± 9.09	65.82 ± 11.33	48.19 ± 11.87	<0.001***
Male/n (%)^b^	22 (36.7%)	20 (33.3%)	17 (32.1%)	0.196
Female n (%)^b^	38 (63.3%)	40 (66.7%)	36 (67.9%)	
BMI^a^	23.53 ± 4.66	23.33 ± 3.79	22.66 ± 3.96	0.353
Course of disease (month)^c^	66.00 (50.25-78.75)	63.00 (48.25-77.00)	–	0.466
Traditional risk factors				
Smoking n (%)^b^	34 (56.7%)	30 (50.0%)	29 (54.7)	0.323
Drinking n (%)^b^	15 (25.0%)	10 (16.7%)	6 (11.3%)	<0.001***
Hypertension n (%)^b^	14 (23.3%)	13 (21.7%)	–	0.827
Diabetes n (%)^b^	11 (18.3%)	6 (10.0%)	–	0.191
Clinical cardiologic diagnosis				
Stable angina n (%)^b^	32 (53.3%)	–	–	–
Unstable angina n (%)^b^	28 (46.7%)	–	–	–
Current use of medication				
NSAIDs n (%)^b^	43 (71.7%)	46 (76.7%)	–	0.532
csDMARDs n (%)^b^	45 (75.0%)	47 (78.3%)	–	0.666
bDMARDs n (%)^b^	1 (1.7%)	2 (3.3%)	–	0.559
GC n (%)^b^	40 (66.7%)	40 (68.3%)	–	0.845
Statins n (%)^b^	58 (96.7%)	–	–	–
Anti-platelet drug n (%)^b^	57 (95.0%)	–	–	–
Beta-blockers n (%)^b^	18 (30.0%)	–	–	–
ACEI/ARBs n (%)^b^	5 (8.3%)	–	–	–
Coronary-expansion drugs n (%)^b^	17 (28.3%)	–	–	–
Laboratory Characteristics				
DAS 28^c^	6.58 (6.10-7.04)	6.52 (6.17-6.86)	–	0.442
RF (U/mL)^c^	70.70 (48.93-106.50)	59.62 (40.00-98.70)	–	0.436
Anti-CCP (U/mL)^c^	641.17 (323.34-841.00)	525.60 (278.95-780.35)	–	0.228
ESR (mm/h)^c^	38.00 (18.00-69.00)	25.50 (12.75-81.25)	9.00 (7.00-14.00)	<0.001***
CRP (mg/L)^c^	12.56 (3.28-42.90)	3.26 (1.52-9.51)	1.32 (0.85-2.32)	<0.001***
Complete blood count				
WBC (*10^9^/L)^c^	6.97 (5.54-8.23)	7.19 (5.28-8.57)	6.74 (4.78-7.60)	0.072
RBC (*10^12^/L)^c^	4.14 (3.78-4.57)	4.21 (3.76-4.60)	4.23 (3.98-4.50)	0.959
Hb (g/L)^c^	121.50 (111.50-136.75)	123.50 (107.25-138.00)	123.00 (116.50-129.00)	0.897
PLT (*10^9^/L)^c^	266.00 (203.25-322.50)	252.50 (204.25-295.25)	245.00 (158.50-287.00)	0.093
LY (*10^9^/L)^c^	1.51 (1.06-1.90)	1.44 (1.18-1.75)	1.26 (1.15-1.70)	0.521
MONO (*10^9^/L)^c^	0.51 (0.35-0.66)	0.45 (0.36-0.57)	0.43 (0.37-0.48)	0.079
NEUT (*10^9^/L)^c^	5.26 (3.99-6.47)	4.70 (3.66-6.28)	4.91 (3.48-5.43)	0.050
Liver Function Test				
ALT (U/L)^c^	15.85 (10.23-19.18)	13.60 (9.53-18.70)	13.80 (9.40-17.25)	0.218
AST (U/L)^c^	18.20 (13.85-22.65)	18.65 (15.85-20.90)	20.08 (16.30-22.65)	0.991
TBIL (μmol/L)^c^	10.30 (8.58-13.45)	9.80 (8.03-13.55)	9.70 (8.10-11.95)	0.336
DBIL (μmol/L)^c^	2.10 (1.70-3.25)	2.10 (1.50-2.60)	2.10 (1.50-2.95)	0.481
IBIL (μmol/L)^c^	8.15 (6.83-11.08)	7.75 (6.57-10.95)	7.70 (6.10-9.25)	0.284
CHOL (mmol/L)^c^	3.91 (3.24-4.75)	3.92 (3.21-4.43)	3.85 (3.42-4.41)	0.896
TG (mmol/L)^c^	1.13 (0.87-1.66)	1.17 (0.90-1.51)	1.02 (0.82-1.31)	0.044*
HDL-C (mmol/L)^c^	1.24 (1.06-1.51)	1.08 (0.90-1.36)	1.28 (1.15-1.44)	<0.001***
LDL-C (mmol/L)^c^	3.11 (2.08-4.44)	1.53 (1.18-2.11)	1.63 (1.23-2.13)	<0.001***
Kidney Function Test				
BUN (mmol/L)^c^	5.35 (4.33-6.40)	5.80 (4.70-6.90)	5.30 (4.35-6.30)	0.357
Cr (μmol/L)^c^	56.00 (47.25-62.00)	58.00 (51.75-65.00)	54.00 (47.50-60.50)	0.081
UA (μmol/L)^c^	252.50 (188.75-308.00)	259.00 (215.00-313.25)	250.00 (221.00-276.00)	0.197
Immunoglobulin				
IgA (g/L)^c^	2.89 (2.58-4.07)	2.08 (1.64-2.84)	1.92 (1.22-2.37)	<0.001***
IgG (g/L)^c^	12.57 (11.37-14.80)	11.38 (10.28-13.07)	11.29 (9.23-14.38)	0.008**
IgM (g/L)^c^	1.01 (0.69-1.54)	1.09 (0.61-1.55)	0.99 (0.62-1.26)	0.366

a Date with mean ± standard deviation.

b Data with number (n)/percentage (%).

c Date with median and 25th and 75th percentiles.

BMI, Body mass index; NSAIDs, Nonsteroidal antiinflammatory drugs; csDMARDs, Conventional synthetic disease-modifying antirheumatic drugs; bDMARD, Biological disease-modifying antirheumatic drug; GC, Glucocorticoid; ACEI, Angiotensin-converting enzyme inhibitors; ARB, Angiotensin receptor blockers; DAS28, Disease activity score 28; RF, Rheumatoid factor; Anti-CCP, Anti-cycliccitrullinated peptide; ESR, Erythrocyte sedimentation rate; CRP, C-reactive protein; WBC, White blood cell; RBC, Red blood cell; Hb, Hemoglobin; PLT, Platelet; LY, Lymphocyte; MONO, Monocyte; NEUT, Neutrophils; ALT, Alanine transaminase; AST, Aspartic transaminase; TBIL, Total bilirubin; DBIL, Direct bilirubin; IBIL, Indirect bilirubin; CHOL, Cholesterol; TG, Triglycerides; HDL-C, High density lipoprotein cholesterol; LDL-C, Low-density lipoprotein cholesterol; BUN, Blood urea nitrogen; Cr, Creatinine; UA, Uric acid; IgA, Immunoglobulin A; IgG, Immunoglobulin G; IgM, Immunoglobulin M.*P<0.05, **P<0.01, ***P<0.001.

Compared to the HC group, both the RA-obstructive CAD and RA-non-obstructive CAD groups exhibited significantly elevated ESR and CRP levels, with the RA-obstructive CAD group showing notably higher CRP levels than the RA-non-obstructive CAD group. Regarding serum lipoproteins, triglyceride (TG) levels were lower and high-density lipoprotein cholesterol (HDL-C) levels were higher in the HC group. The RA-obstructive CAD group displayed higher levels of low-density lipoprotein cholesterol (LDL-C) and HDL-C compared to the RA-non-obstructive CAD group. Furthermore, serum immunoglobulin A (IgA) and immunoglobulin G (IgG) levels were lower in the HC group, while the RA-obstructive CAD group exhibited elevated IgA and IgG levels relative to the RA-non-obstructive CAD group. No significant differences were observed in other parameters (**Supplementary Table 1**).

### 3.2 Differences in peripheral blood lymphocyte subsets and CD4+ T cell levels between the RA-obstructive CAD and RA-non-obstructive CAD groups

**Figure 1:**
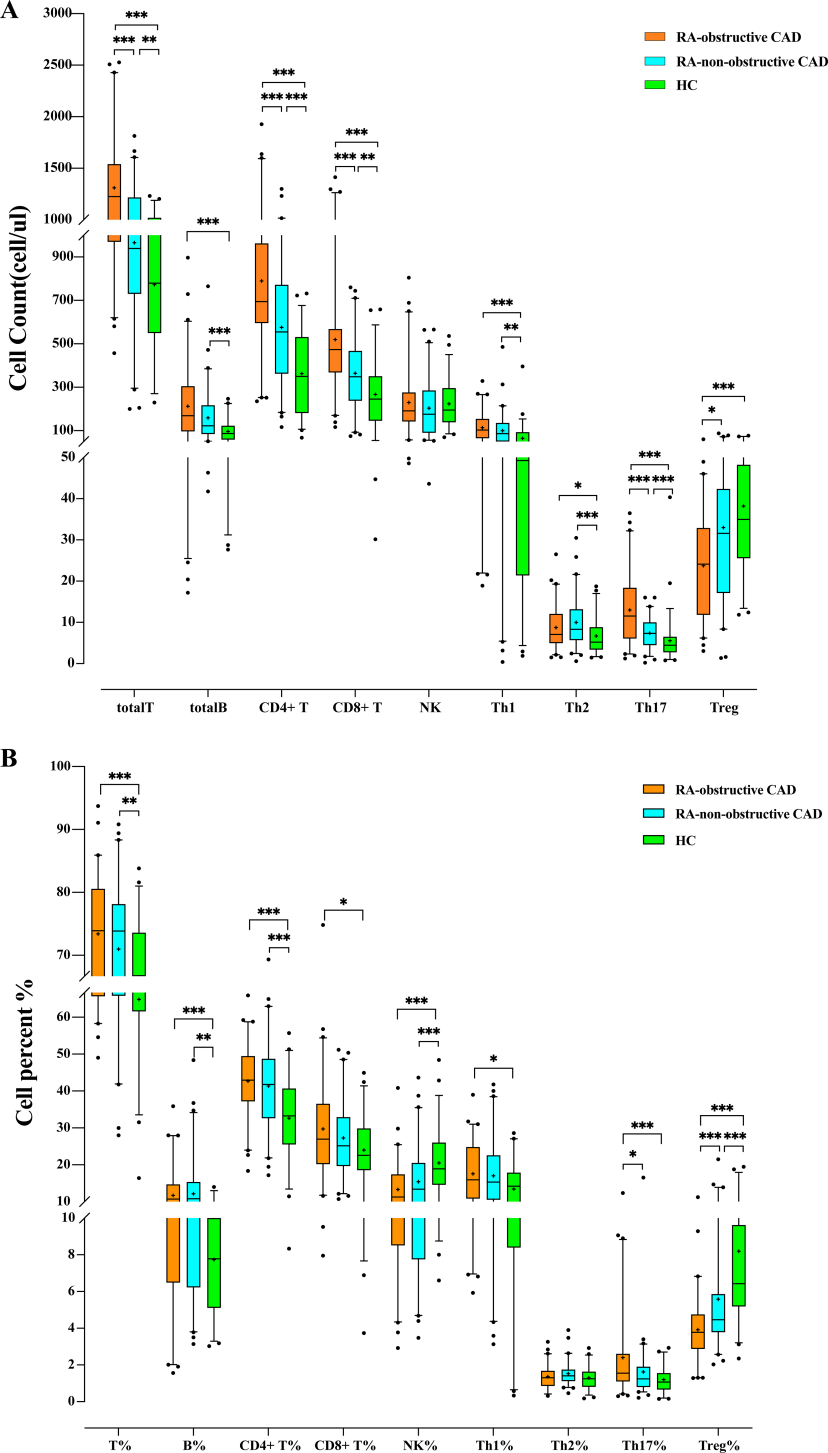
**(A)** Comparison of peripheral blood lymphocyte subsets and CD4+T cell counts among each study group. **(B)** Comparison of the proportion of peripheral blood lymphocyte subsets and CD4+ T cells among each study group. (*p < 0.05, **p < 0.01, ***p < 0.001).

We compared the quantities and percentages of peripheral blood lymphocyte subsets and CD4+ T cell subsets between the two RA groups and the HC group. Compared with the HC group, the total T cell count (P<0.001) and percentage (P<0.001), total B cell count (P<0.001) and percentage (P<0.001), CD4+ T cell count (P<0.001) and percentage (P<0.001), CD8+ T cell count (P<0.001) and percentage (P=0.032), Th1 cell count (P<0.001), Th2 cell count (P=0.003), Th17 cell count (P<0.001) and percentage (P<0.001), Th1/Th2 ratio (P=0.006), and Th17/Treg ratio (P<0.001) were all significantly elevated. The NK cell percentage (P<0.001) and Treg cell count (P<0.001) and percentage (P<0.001) in the HC group were significantly higher than those in both RA groups (**Tables 2A, B**; **Figures 1A, B**).

**Table 2:** Absolute counts and proportions of peripheral blood lymphocytes in the RA-Obstructive CAD, RA-Non-Obstructive CAD, and HC Groups.

(A)	RA-Obstructive CAD (n=60)	RA-Non-Obstructive CAD (n=60)	HC (n=53)	*p*
totalT (cells/μL)	1225.71 (970.23-1538.75)	939.48 (730.65-1216.34)	778.66 (548.75-1018.14)	<0.001***
totalB (cells/μL)	169.29 (96.90-304.63)	122.35 (85.17-216.37)	86.36 (58.00-121.82)	<0.001***
CD4+ T (cells/μL)	694.29 (595.83-962.43)	555.07 (362.68-771.16)	349.27 (180.89-530.56)	<0.001***
CD8+ T (cells/μL)	473.67 (368.32-568.12)	348.34 (238.52-467.11)	244.50 (145.35-349.55)	<0.001***
NK (cells/μL)	191.68 (142.79-275.42)	175.55 (90.61-284.70)	194.49 (138.39-295.09)	0.37
Th1 (cells/μL)	103.05 (65.18-154.31)	86.09 (50.35-135.44)	49.24 (21.37-92.39)	<0.001***
Th2 (cells/μL)	7.08 (5.00-12.02)	8.33 (5.70-13.17)	5.19 (3.35-8.79)	0.003**
Th17 (cells/μL)	11.53 (6.08-18.38)	7.33 (4.51-9.99)	4.43 (2.72-6.47)	<0.001***
Treg (cells/μL)	23.15 (11.85-32.90)	31.61 (17.15-42.36)	34.93 (25.53-48.16)	<0.001***
(B)	RA-Obstructive CAD (n=60)	RA-Non-Obstructive CAD (n=60)	HC (n=53)	*p*
T%	73.90 (65.68-80.55)	73.87 (65.81-78.14)	66.75 (61.62-73.59)	<0.001***
B%	10.69 (6.49-14.69)	10.78 (6.23-15.34)	7.78 (5.11-10.00)	<0.001***
CD4+ T%	42.93 (37.19-49.27)	41.78 (32.64-48.73)	33.23 (25.56-40.68)	<0.001***
CD8+ T%	26.97 (20.21-36.54)	25.15 (19.68-32.89)	22.56 (18.55-29.83)	0.032*
CD4+ T/CD8+ T	1.67 (1.11-2.25)	1.61 (1.11-2.18)	1.37 (1.09-1.90)	0.338
NK%	11.28 (8.52-17.39)	13.38 (7.75-20.49)	18.88 (14.60-26.01)	<0.001***
Th1%	15.95 (10.85-24.82)	15.31 (10.55-22.54)	14.21 (8.40-17.87)	0.081
Th2%	1.30 (0.86-1.67)	1.41 (1.12-1.75)	1.25 (0.81-1.64)	0.155
Th17%	1.55 (1.10-2.61)	1.25 (0.80-1.90)	1.07 (0.67-1.55)	<0.001***
Treg%	3.78 (2.88-4.75)	4.47 (3.79-5.86)	6.43 (5.19-9.62)	<0.001***
Th1/Th2	13.03 (8.10-20.80)	10.32 (6.23-16.88)	9.24 (5.87-13.35)	0.006**
Th17/Treg	0.40 (0.30-1.03)	0.25 (0.17-0.40)	0.20 (0.11-0.31)	<0.001***

Date with median and 25th and 75th percentiles.

T, T lymphocyte; B, B lymphocyte; NK, Natural killer cell; Th1, T-helper 1 cells; Th2, T-helper 2 cells; Th17, T-helper17 cells; Treg, Regulatory T cells. *P<0.05, **P<0.01, ***P<0.001.

Additionally, we compared the peripheral blood lymphocyte subsets and CD4+ T cell subsets between the RA-obstructive CAD and RA-non-obstructive CAD groups. We found that the total T cell count (P<0.001), CD4+ T cell count (P<0.001), CD8+ T cell count (P<0.001), Th17 cell count (P<0.001) and percentage (P=0.019), Th1/Th2 ratio (P=0.032), and Th17/Treg ratio (P<0.001) were significantly higher in the RA-obstructive CAD group, while the Treg cell count (P=0.011) and percentage (P<0.001) were lower compared to the RA-non-obstructive CAD group (**Tables 2A, B**; **Figures 1A, B**; **Supplementary Table 2**).

### 3.3 Differences in cytokine levels between the RA-obstructive CAD and RA-non-obstructive CAD groups

**Figure 2:**
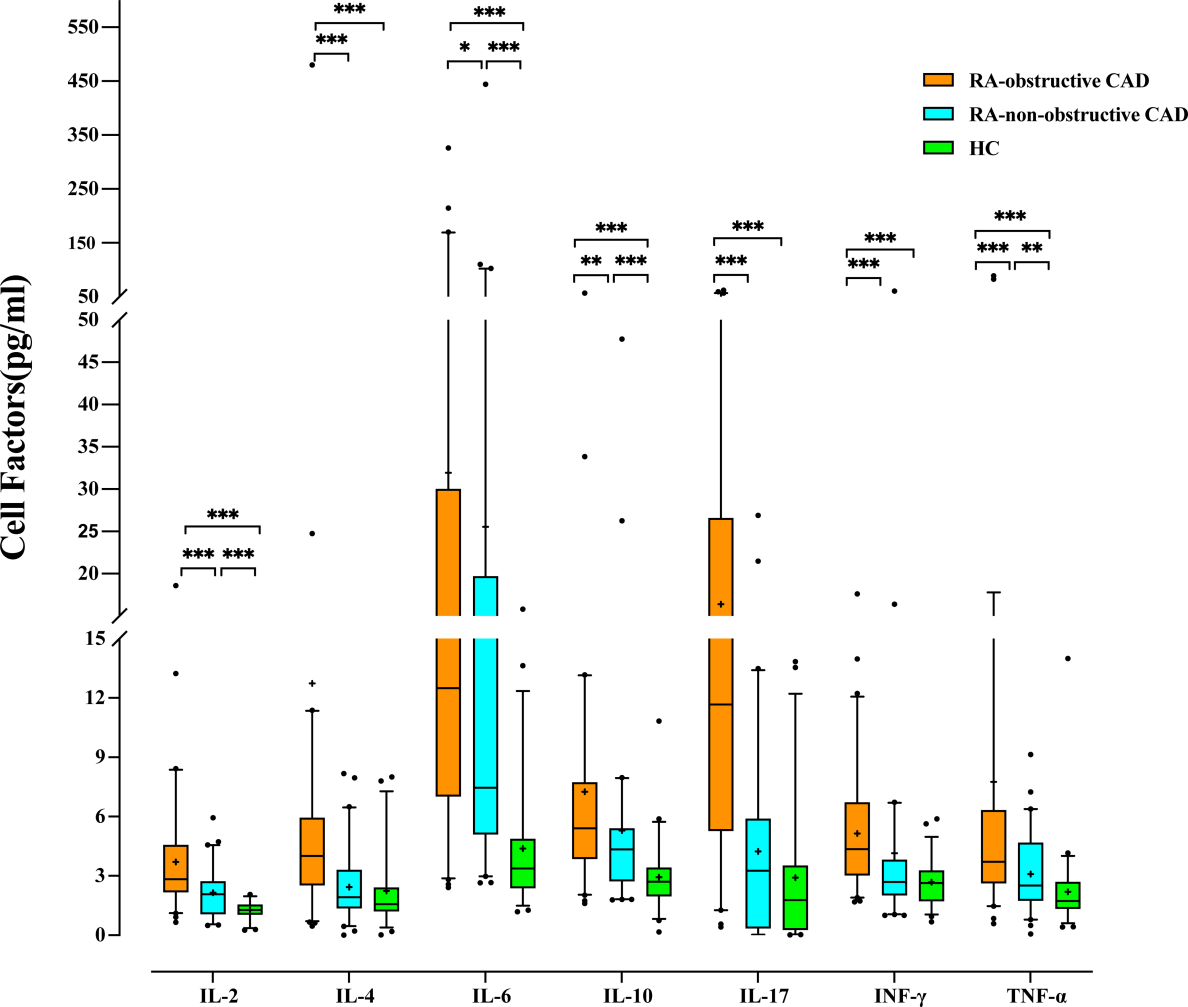
Comparison of Cytokine levels among each study group. (*p < 0.05, **p < 0.01, ***p < 0.001).

We compared cytokine levels between the RA groups and the HC group. Compared to the HC group, RA patients exhibited significantly elevated levels of IL-2 (P<0.001), IL-4 (P<0.001), IL-6 (P<0.001), IL-10 (P<0.001), IL-17 (P<0.001), IFN-γ (P<0.001), and TNF-α (P<0.001) (**Table 3**; **Figure 2**; **Supplementary Table 2**).

**Table 3:** Cytokine Levels in Peripheral Blood of the RA-obstructive CAD, RA-non-obstructive CAD, and HC Groups.

	RA-obstructive CAD (n=60)	RA-non-obstructive CAD (n=60)	HC (n=53)	*p*
IL-2	2.83 (2.18-4.56)	2.07 (1.08-2.72)	1.28 (1.05-1.54)	<0.001***
IL-4	4.01 (2.53-5.93)	1.93 (1.37-3.30)	1.57 (1.23-2.41)	<0.001***
IL-6	12.21 (6.99-29.91)	7.47 (5.11-19.68)	3.38 (2.39-4.87)	<0.001***
IL-10	5.41 (3.86-7.72)	4.34 (2.74-5.40)	2.71 (1.98-3.42)	<0.001***
IL-17	11.67 (5.28-26.56)	3.26 (0.36-5.88)	1.78 (0.28-3.52)	<0.001***
IFN-γ	4.35 (3.03-6.72)	2.69 (2.02-3.81)	2.64 (1.74-3.27)	<0.001***
TNF-α	3.72 (2.64-6.31)	2.51 (1.76-4.67)	1.73 (1.35-2.69)	<0.001***

Date with median and 25th and 75th percentiles.

IL-2, Interleukin-2; IL-4, Interleukin-4; IL-6, Interleukin-6; IL-10, Interleukin-10; IL-17, Interleukin-17; INF-γ, Interferon-γ; TNF-α, Tumor necrosis factor-α.***P<0.001.

Additionally, we compared cytokine levels between the RA-obstructive CAD and RA-non-obstructive CAD groups. The RA-obstructive CAD group exhibited significantly higher levels of cytokines, including IL-2 (P<0.001), IL-4 (P<0.001), IL-6 (P=0.037), IL-10 (P=0.001), IL-17 (P<0.001), IFN-γ (P<0.001), and TNF-α (P<0.001) (**Table 3**; **Figure 2**; **Supplementary Table 2**).

### 3.4 Development of a personalized prediction model for RA patients with obstructive CAD

**Figure 3:**
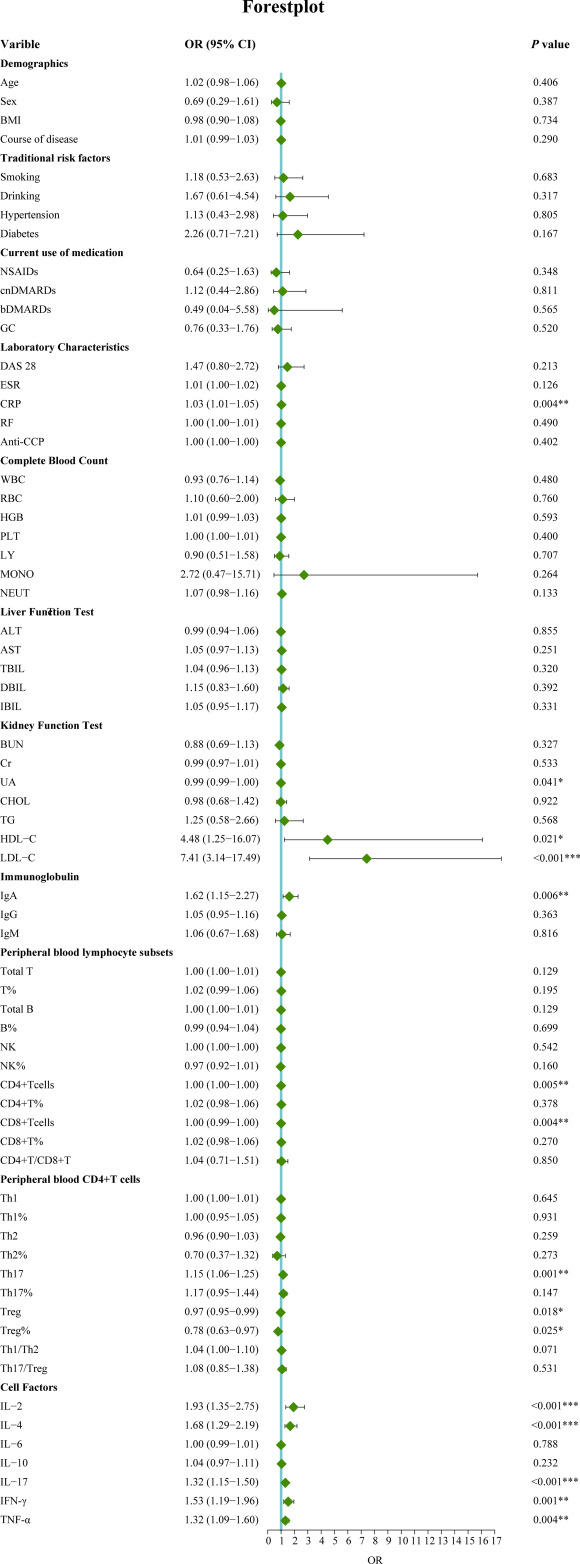
Univariate logistic regression analysis of factors associated with obstructive CAD in RA patients. (*p < 0.05, **p < 0.01, ***p < 0.001). OR, Odds ratio; 95%CI, 95% confidence interval; BMI, Body mass index; NSAIDs, Nonsteroidal antiinflammatory drugs; csDMARDs, Conventional synthetic disease-modifying antirheumatic drugs; bDMARD, Biological disease-modifying antirheumatic drug; GC, Glucocorticoid; DAS28, Disease activity score 28; ESR, Erythrocyte sedimentation rate; CRP, C-reactive protein; RF, Rheumatoid factor; Anti-CCP, Anti-cycliccitrullinated peptide; WBC, White blood cell; RBC, Red blood cell; Hb, Hemoglobin; PLT, Platelet; LY, Lymphocyte; MONO, Monocyte; NEUT, Neutrophils; ALT, Alanine transaminase; AST, Aspartic transaminase; TBIL, Total bilirubin; DBIL, Direct bilirubin; IBIL, Indirect bilirubin; CHOL, Cholesterol; TG, Triglycerides; HDL-C, High density lipoprotein cholesterol; LDL-C, Low-density lipoprotein cholesterol; BUN, blood urea nitrogen; Cr, Creatinine; UA, Uric acid; IgA, Immunoglobulin A; IgG, Immunoglobulin G; IgM, Immunoglobulin M. T, T lymphocyte; B, B lymphocyte; NK, Natural killer cell; Th1, T-helper 1 cells; Th2, T-helper 2 cells; Th17, T-helper17 cells; Treg, Regulatory T cells.IL-2, Interleukin-2; IL-4, Interleukin-4; IL-6, Interleukin-6; IL-10, Interleukin-10; IL-17, Interleukin-17; INF-γ, Interferon-γ; TNF-α, Tumor necrosis factor-α.

**Figure 4:**
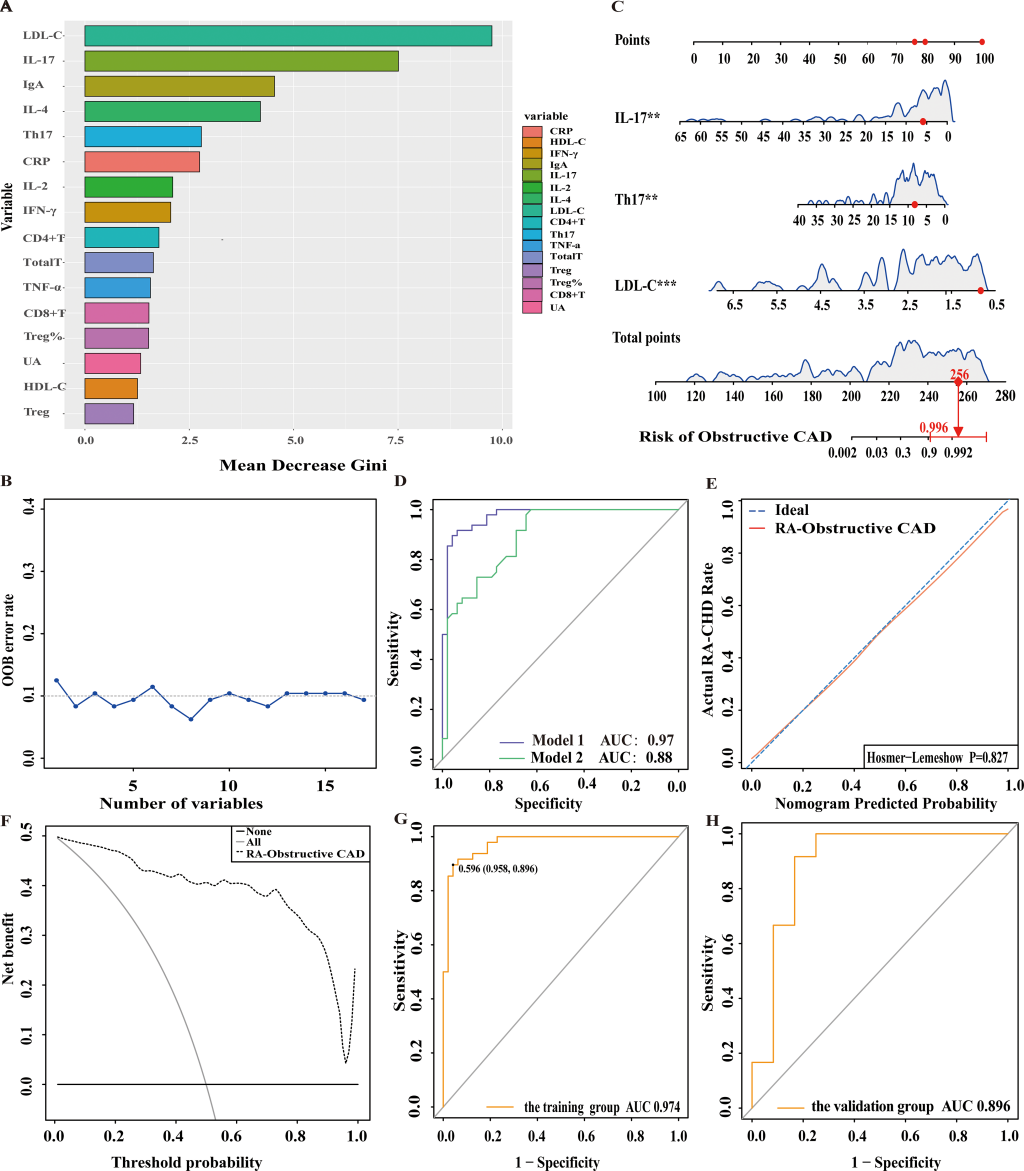
Nomogram for predicting and assessing the risk of obstructive CAD in RA patients. **(A)** Ranking of risk factors for obstructive CAD development in RA patients by importance. **(B)** The relationship between the number of predictive indicators for obstructive CAD and the out-of-bag (OOB) error rate. **(C)** Nomogram for predicting the risk of obstructive CAD in RA patients. **(D)** Receiver operating characteristic (ROC) curves for predicting obstructive CAD risk in RA patients. Model 1: Nomogram incorporating LDL-C, Th17, and IL-17; Model 2: Nomogram incorporating LDL-C as the sole predictor. **(E)** Calibration curve for obstructive CAD risk prediction in RA patients. **(F)** DCA for predicting obstructive CAD risk in RA patients. **(G)** ROC curve of the nomogram for predicting obstructive CAD risk in the training cohort. **(H)** ROC curve of the nomogram for validating obstructive CAD risk in the external validation cohort.

In this study, 120 RA patients were randomly assigned to a training group (96 patients) and a validation group (24 patients). No significant differences were observed between the two groups in terms of clinical data, laboratory results, and peripheral blood lymphocyte subsets (P>0.05). To identify distinguishing factors between the RA-obstructive CAD and RA-non-obstructive CAD groups, univariate logistic regression analysis was performed on clinical characteristics, laboratory markers, peripheral blood lymphocyte subsets, CD4+ T cell subsets, and cytokine levels in the training group. The analysis revealed significant differences in CRP, uric acid (UA), HDL-C, LDL-C, IgA, CD4+ T cells, CD8+ T cells, Th17, Treg, Treg ratio, IL-2, IL-4, IL-17, IFN-γ, and TNF-α between the two groups (P<0.05)(**Figure 3**). These statistically significant variables were then input into a random forest model, and the importance of each variable was ranked based on the MDG (**Figure 4A**). Based on the ranking, random forest regression analysis was conducted, revealing that selecting 8 variables minimized the OBB (**Figure 4B**). Subsequently, the top 8 variables (LDL-C, IL-17, IgA, IL-4, Th17, CRP, IL-2, and IFN-γ) were entered into a stepwise multivariate logistic regression analysis. The results identified LDL-C, IL-17, and Th17 as significantly differing factors (**Table 4**). These variables were then incorporated into a risk prediction model for RA-obstructive CAD, resulting in the construction of nomogram prediction model 1 (**Figure 4C**). Considering that Th17 and IL-17 are not traditional CAD risk factors, they were excluded from the model, and prediction model 2 was subsequently developed. The predictive performance of both models was then compared.

**Table 4:** Multivariate logistic regression analysis of factors associated with obstructive CAD in RA patients.

	Model 1		Model 2		*p*
	OR (95% CI)	*p*	OR (95% CI)	*p*	
LDL-C	11.28 (3.59-75.97)	0.003**	7.41 (3.14-17.49)	<0.001***	0.003**
Th17	1.28 (1.09-1.60)	0.003**	–	–	
IL-17	1.28 (1.07-1.63)	0.006**	–	–	
AUC	0.97 (0.94-1.00)		0.88 (0.81-0.94)		

OR, Odds ratio; 95%CI, 95% Confidence interval; LDL-C, Low-density lipoprotein cholesterol; Th17, T-helper17 cells; IL-17, Interleukin-17; AUC, Area under the ROC curve. **p<0.01, ***P<0.001.

### 3.5 Validation of the nomogram

After comparing the two prediction models, we found that model 1 had an AUC of 0.97, while model 2 had an AUC of 0.88, with a significant difference between them (p=0.003), indicating that model 1 outperformed model 2 in terms of discriminative ability (**Figure 4D**). To further evaluate the model’s discriminatory power, we plotted the ROC curves for both groups. The results showed an AUC of 0.974 for the training group (**Figure 4G**) and an AUC of 0.896 for the validation group (**Figure 4H**). In both groups, the AUC exceeded 0.75, demonstrating the model’s strong discriminative ability.

Additionally, we assessed the model’s calibration by plotting calibration curves, which showed a high degree of alignment between the predicted and observed curves, suggesting robust calibration performance. Further, the Hosmer-Lemeshow test yielded a p-value of 0.827 (P>0.05) (**Figure 4E**), providing additional support for the model’s excellent fit.

### 3.6 Clinical use

We then generated the DCA, which showed that the cut-off value (59.6%) obtained from the ROC analysis (**Figure 4G**) lies within the threshold probability range of the DCA curve. Further analysis indicated that, when the threshold probability for diagnosing obstructive CAD in RA patients was set at 59.6%, approximately 40 out of 100 RA patients at risk for obstructive CAD diagnosed using this model would benefit, without causing unnecessary harm to other patients (**Figure 4F**).

## 4 Discuss

In this study, a nomogram model developed from electronic medical record (EMR) data integrates traditional LDL-C with emerging immune biomarkers, Th17 and IL-17, to provide a novel approach for assessing obstructive CAD risk in patients with RA. The findings reveal significant differences in multiple clinical and laboratory parameters, immune cell composition, and cytokine levels between the RA-obstructive CAD and RA-non-obstructive CAD groups, particularly in the elevated inflammatory markers CRP, Th17 cells, and IL-17. These results align with existing literature, further emphasizing the close link between immune dysregulation in RA patients and heightened cardiovascular risk (25, 26).

Compared to traditional cardiovascular risk assessment models, our proposed model, based on immune biomarkers, places greater emphasis on the evaluation of immune-related risk factors. Although classical tools, such as the Framingham Risk Score, are widely used for cardiovascular disease risk prediction (27), these models primarily rely on conventional factors such as lipid levels and blood pressure, and fail to adequately account for the unique immune-inflammatory state in RA patients. RA patients often exist in a state of chronic low-grade inflammation, which not only exacerbates joint damage but also significantly elevates the risk of cardiovascular disease. In this context of immune dysregulation, the predictive capability of traditional models may be limited.This study demonstrates that elevated levels of LDL-C, IL-17, and Th17 cells are closely associated with the occurrence of obstructive CAD in RA patients, highlighting the potential value of these immune biomarkers in assessing the risk of RA-obstructive CAD. Although a direct comparison with traditional risk assessment models was not conducted in this study, the identification of the unique role of immune biomarkers may help address the limitations of conventional models. Incorporating immune biomarkers into cardiovascular risk assessment could provide a more comprehensive tool for clinical use, particularly in the RA patient population, thereby enhancing prediction accuracy.

In this study, immune cell analysis revealed that in the RA-obstructive CAD group, T cells, CD4+ T cells, CD8+ T cells, Th17 cells, and the Th1/Th2 and Th17/Treg ratios were significantly elevated, while the number of Treg cells was reduced. These findings suggest that immune imbalance may play a promoting role in the progression of RA-obstructive CAD. Additionally, we observed increased levels of various pro-inflammatory cytokines (such as IL-2, IL-4, IL-6, IL-10, IL-17, IFN-γ, and TNF-α) in the RA-obstructive CAD group, which are closely associated with sustained inflammatory responses and exacerbated cardiovascular damage. Compared to healthy controls, both the RA-non-obstructive CAD and RA-obstructive CAD groups exhibited significantly elevated ESR and CRP levels, with particularly higher CRP levels in the RA-obstructive CAD group. These findings further confirm the elevated systemic inflammation in RA-obstructive CAD patients, which may contribute to the increased incidence of cardiovascular disease.

Th17 cells and their secreted cytokine IL-17 play a crucial role in the inflammatory response and the development of atherosclerosis. Previous studies have shown that IL-17 is closely associated with atherosclerosis (28), particularly in immune-mediated cardiovascular diseases. Th17 cells secrete cytokines such as IL-17, which, by releasing chemokines like CXCL1, CXCL2, and CXCL8, recruits neutrophils and monocytes to sites of atherosclerotic lesions. IL-17 also stimulates macrophages to produce pro-inflammatory cytokines such as IL-6, TNF-α, and IL-1β (29–31). Furthermore, IL-17 can promote the production of matrix metalloproteinases (MMPs) in fibroblasts, endothelial cells, and epithelial cells (31, 32). Through these mechanisms, IL-17 enhances endothelial inflammation, increases arterial wall permeability, and stimulates smooth muscle cell proliferation, which may play a key role in the pathogenesis of atherosclerosis. In contrast, regulatory Treg cells are essential for maintaining immune tolerance and suppressing excessive immune responses. However, in RA-obstructive CAD patients, impaired Treg function may lead to dysregulated inflammatory responses. The reduction of Treg cells is closely linked to the increase in Th17 cells, driving the progression of RA and related cardiovascular diseases (25). Our data support this notion, demonstrating elevated Th17 cells and IL-17 in RA-obstructive CAD patients, which may exacerbate the progression of atherosclerosis and serve as potential targets for immunotherapy.

To further explore the role of these immune biomarkers in RA-obstructive CAD, we conducted univariate logistic regression analysis to compare clinical characteristics, immune features, and cytokine levels between RA-non-obstructive CAD and RA-obstructive CAD patients, followed by random forest analysis to identify key variables. Multivariate logistic regression analysis revealed that LDL-C, IL-17, and Th17 are independent risk factors for RA-obstructive CAD. Based on these findings, two nomogram models were constructed. Model 1 (including LDL-C, IL-17, and Th17) outperformed Model 2 in predicting RA-obstructive CAD risk (AUC = 0.97 vs. AUC = 0.88), validating the importance of immune biomarkers in RA-obstructive CAD risk assessment. Notably, Th17 cells and IL-17 not only serve as immunological markers for RA but also play a significant role in the immune mechanisms underlying cardiovascular diseases.

Further validation through ROC curve and decision curve analysis (DCA) confirmed the practical applicability of the prediction model in clinical settings. The model demonstrated an AUC greater than 0.75 in both the training and validation cohorts, indicating strong discriminatory power for clinical risk prediction of RA-obstructive CAD. The optimal cutoff value was determined using the Youden index (Youden index = sensitivity + specificity - 1), with the maximum index corresponding to a cutoff of 59.6%. When this threshold was applied in DCA, the model showed a high clinical net benefit, suggesting that intervention should be considered when the predicted risk exceeds 59.6%. This model effectively identifies the risk of obstructive CAD in RA patients and supports targeted interventions in high-risk individuals, optimizing treatment strategies while avoiding unnecessary diagnostic and therapeutic risks.

Although this study provides a valuable tool for personalized risk prediction in RA-obstructive CAD, several limitations remain. First, the sample size is relatively small, and the study is based on a single-center design. Future research should involve large-scale multicenter clinical studies to further validate the model’s accuracy and generalizability. Second, while this study highlights the potential value of immune markers in assessing RA-obstructive CAD risk, it did not incorporate other traditional cardiovascular risk factors. Future studies should design larger, more diverse randomized clinical trials or cohort studies that integrate immune markers with traditional cardiovascular risk factors to optimize cardiovascular disease risk assessment and intervention strategies in RA patients. Additionally, further investigation into the biological pathways through which these markers influence CAD is needed. Third, this study did not compare the proposed model with existing cardiovascular risk assessment tools. Future research should involve randomized clinical trials or cohort studies to directly compare this model with traditional risk models to assess its clinical applicability and potential for broader use. Fourth, as the nomogram was developed based on a cohort of hospitalized RA patients with high disease activity and predominantly coronary atherosclerosis, future studies should aim to validate the model in asymptomatic RA patients with better clinical control through large-scale multicenter cohort studies. Finally, although IL-17 plays a crucial role in the immune mechanisms of RA-obstructive CAD, its sensitivity and specificity remain relatively low, potentially influenced by other immune factors or external environmental factors. Therefore, future research should focus on improving the accuracy and reliability of IL-17 detection and actively explore more specific immune biomarkers.

In conclusion, the immune biomarker-based risk prediction model proposed in this study offers a novel approach for assessing and managing the risk of RA-obstructive CAD, with significant potential for clinical application. With further validation and optimization, this model is expected to play a key role in personalized treatment and cardiovascular risk management for RA patients.

## Data Availability

The original contributions presented in the study are included in the article/**Supplementary Material**. Further inquiries can be directed to the corresponding authors.
