# Supplementary material for: Development and validation of a nomogram for predicting the risk of obstructive coronary artery disease in rheumatoid arthritis patients based on LDL-C, Th17 cells, and IL-17
Source: Front Immunol. 2024 Dec 17;15:1493182. doi: 10.3389/fimmu.2024.1493182 (PMC11685205; doi:10.3389/fimmu.2024.1493182)
Supplement: Supplementary file 1 [file Table1.docx]

| **Supplementary Table 1.** Clinical characteristics of the RA-Obstructive CAD and RA-Non-Obstructive CAD Groups. | | | |
| --- | --- | --- | --- |
|  | **RA-Obstructive CAD(n=60)** | **RA-Non-Obstructive CAD(n=60)** | ***P*** |
| Demographics |  |  |  |
| Age(Years)^a^ | 66.92±9.09 | 65.82±11.33 | 0.879 |
| Male n(%)^b^ | 22(36.7%) | 20(33.3%) | 0.702 |
| Female n(%)^b^ | 38(63.3%) | 40(66.7%) |  |
| BMI^a^ | 23.53±4.66 | 23.33±3.79 | 0.280 |
| Course of disease (month)^c^ | 66.00(50.25-78.75) | 63.00(48.25-77.00) | 0.466 |
| Traditional risk factors |  |  |  |
| Smoking n(%)^b^ | 34(56.7%) | 30(50.0%) | 0.464 |
| Drinking n(%)^b^ | 15(25.0%) | 10(16.7%) | 0.261 |
| Hypertension n(%)^b^ | 14(23.3%) | 13(21.7%) | 0.827 |
| Diabetes n(%)^b^ | 11(18.3%) | 6(10.0%) | 0.191 |
| Current use of medication |  |  |  |
| NSAIDs n (%)^b^ | 43(71.7%) | 46(76.7%) | 0.532 |
| csDMARDs n (%)^b^ | 45(75.0%) | 47(78.3%) | 0.666 |
| bDMARDs n (%)^b^ | 1(1.7%) | 2(3.3%) | 0.559 |
| GC n (%)^b^ | 40(66.7%) | 40(68.3%) | 0.845 |
| Laboratory Characteristics |  |  |  |
| DAS 28^c^ | 6.58(6.10-7.04) | 6.52(6.17-6.86) | 0.442 |
| RF (U/mL)^c^ | 70.70(48.93-106.50) | 59.62(40.00-98.70) | 0.436 |
| Anti-CCP (U/mL)^c^ | 641.17(323.34-841.00) | 525.60(278.95-780.35) | 0.228 |
| ESR(mm/h)^c^ | 38.00(18.00-69.00) | 25.50(12.75-81.25) | 0.062 |
| CRP(mg/L)^c^ | 12.56(3.28-42.90) | 3.26(1.52-9.51) | <0.001*** |
| Complete blood count |  |  |  |
| WBC(*10^9^/L)^c^ | 6.97(5.54-8.23) | 7.19(5.28-8.57) | 0.805 |
| RBC(*10^12^/L)^c^ | 4.14(3.78-4.57) | 4.21(3.76-4.60) | 0.971 |
| Hb(g/L)^c^ | 121.50(111.50-136.75) | 123.50(107.25-138.00) | 0.923 |
| PLT(*10^9^/L)^c^ | 266.00(203.25-322.50) | 252.50(204.25-295.25) | 0.492 |
| LY(*10^9^/L)^c^ | 1.51(1.06-1.90) | 1.44(1.18-1.75) | 0.830 |
| MONO(*10^9^/L)^c^ | 0.51(0.35-0.66) | 0.45(0.36-0.57) | 0.278 |
| NEUT(*10^9^/L)^c^ | 5.26(3.99-6.47) | 4.70(3.66-6.28) | 0.195 |
| Liver Function Test |  |  |  |
| ALT(U/L)^c^ | 15.85(10.23-19.18) | 13.60(9.53-18.70) | 0.378 |
| AST(U/L)^c^ | 18.20(13.85-22.65) | 18.65(15.85-20.90) | 0.883 |
| TBIL(μmol/L)^c^ | 10.30(8.58-13.45) | 9.80(8.03-13.55) | 0.357 |
| DBIL(μmol/L)^c^ | 2.10(1.70-3.25) | 2.10(1.50-2.60) | 0.249 |
| IBIL(μmol/L)^c^ | 8.15(6.83-11.08) | 7.75(6.57-10.95) | 0.517 |
| TC(mmol/L)^c^ | 3.91(3.24-4.75) | 3.92(3.21-4.43) | 0.805 |
| TG(mmol/L)^c^ | 1.13(0.87-1.66) | 1.17(0.90-1.51) | 0.900 |
| HDL-C(mmol/L)^c^ | 1.24(1.06-1.51) | 1.08(0.90-1.36) | 0.011* |
| LDL-C(mmol/L)^c^ | 3.11(2.08-4.44) | 1.53(1.18-2.11) | <0.001*** |
| Kidney Function Test |  |  |  |
| BUN(mmol/L)^c^ | 5.35(4.33-6.40) | 5.80(4.70-6.90) | 0.389 |
| (Continued) | | | |
| Table 1 Continued | | | |
| Cr(μmol/L)^c^ | 56.00(47.25-62.00) | 58.00(51.75-65.00) | 0.314 |
| UA(μmol/L)^c^ | 252.50(188.75-308.00) | 259.00(215.00-313.25) | 0.239 |
| Immunoglobulin |  |  |  |
| IgA(g/L)^c^ | 2.89(2.58-4.07) | 2.08(1.64-2.84) | <0.001*** |
| IgG(g/L)^c^ | 12.57(11.37-14.80) | 11.38(10.28-13.07) | 0.011* |
| IgM(g/L)^c^ | 1.01(0.69-1.54) | 1.09(0.61-1.55) | 0.952 |

a Date with mean±standard deviation

b Data with number (n)/percentage (%)

C Date with median and 25th and 75th percentiles

BMI: Body mass index; NSAIDs, Nonsteroidal antiinflammatory drugs; csDMARDs, Conventional synthetic disease-modifying antirheumatic drugs; bDMARD, Biological disease-modifying antirheumatic drug; GC, Glucocorticoid; ACEI, Angiotensin-converting enzyme inhibitors; ARB, Angiotensin receptor blockers; DAS28, Disease activity score 28; RF, Rheumatoid factor; Anti-CCP, Anti-cycliccitrullinated peptide; ESR, Erythrocyte sedimentation rate; CRP, C-reactive protein; WBC: White blood cell; RBC:Red blood cell; Hb: Hemoglobin; PLT: Platelet; LY: Lymphocyte; MONO: Monocyte; NEUT: Neutrophils; ALT, Alanine transaminase; AST, Aspartic transaminase; TBIL, Total bilirubin; DBIL: Direct bilirubin; IBIL: Indirect bilirubin; CHOL, Cholesterol; TG, Triglycerides; HDL-C, High density lipoprotein cholesterol; LDL-C, Low-density lipoprotein cholesterol; BUN: Blood urea nitrogen; Cr: Creatinine; UA: Uric acid; IgA, Immunoglobulin A; IgG, Immunoglobulin G; IgM, Immunoglobulin M.*P<0.05, ***P<0.001
