# Supplementary material for: Development and validation of a nomogram for predicting the risk of obstructive coronary artery disease in rheumatoid arthritis patients based on LDL-C, Th17 cells, and IL-17
Source: Front Immunol. 2024 Dec 17;15:1493182. doi: 10.3389/fimmu.2024.1493182 (PMC11685205; doi:10.3389/fimmu.2024.1493182)
Supplement: Supplementary file 2 [file Table2.docx]

| **Supplementary Table 2.** Peripheral blood lymphocyte absolute counts and proportions, and cytokine levels in the RA-Obstructive CAD and RA-Non-Obstructive CAD Groups. | | | |
| --- | --- | --- | --- |
|  | **RA-Obstructive CAD(n=60)** | **RA-Non-Obstructive CAD(n=60)** | ***p*** |
| totalT(cells/μL) | 1225.71(970.23-1538.75) | 939.48(730.65-1216.34) | <0.001*** |
| T% | 73.90(65.68-80.55) | 73.87(65.81-78.14) | 0.458 |
| totalB(cells/μL) | 169.29(96.90-304.63) | 122.35(85.17-216.37) | 0.052 |
| B% | 10.69(6.49-14.69) | 10.78(6.23-15.34) | 1.000 |
| CD4+ T(cells/μL) | 694.29(595.83-962.43) | 555.07(362.68-771.16) | <0.001*** |
| CD4+ T% | 42.93(37.19-49.27) | 41.78(32.64-48.73) | 0.443 |
| CD8+ T(cells/μL) | 473.67(368.32-568.12) | 348.34(238.52-467.11) | <0.001*** |
| CD8+ T% | 26.97(20.21-36.54) | 25.15(19.68-32.89) | 0.267 |
| CD4+ T/CD8+ T | 1.67(1.11-2.25) | 1.61(1.11-2.18) | 0.883 |
| NK(cells/μL) | 191.68(142.79-275.42) | 175.55(90.61-284.70) | 0.276 |
| NK% | 11.28(8.52-17.39) | 13.38(7.75-20.49) | 0.376 |
| Th1(cells/μL) | 103.05(65.18-154.31) | 86.09(50.35-135.44) | 0.199 |
| Th1% | 15.95(10.85-24.82) | 15.31(10.55-22.54) | 0.616 |
| Th2(cells/μL) | 7.08(5.00-12.02) | 8.33(5.70-13.17) | 0.180 |
| Th2% | 1.30(0.86-1.67) | 1.41(1.12-1.75) | 0.189 |
| Th17(cells/μL) | 11.53(6.08-18.38) | 7.33(4.51-9.99) | <0.001*** |
| Th17% | 1.55(1.10-2.61) | 1.25(0.80-1.90) | 0.019 * |
| Treg(cells/μL) | 23.15(11.85-32.90) | 31.61(17.15-42.36) | 0.011* |
| Treg% | 3.78(2.88-4.75) | 4.47(3.79-5.86) | <0.001*** |
| Th1/Th2 | 13.03(8.10-20.80) | 10.32(6.23-16.88) | 0.032* |
| Th17/Treg | 0.40(0.30-1.03) | 0.25(0.17-0.40) | <0.001*** |
| IL-2 | 2.83(2.18-4.56) | 2.07(1.08-2.72) | <0.001*** |
| IL-4 | 4.01(2.53-5.93) | 1.93(1.37-3.30) | <0.001*** |
| IL-6 | 12.21(6.99-29.91) | 7.47(5.11-19.68) | 0.047* |
| IL-10 | 5.41(3.86-7.72) | 4.34(2.74-5.40) | 0.001** |
| IL-17 | 11.67(5.28-26.56) | 3.26(0.36-5.88) | <0.001*** |
| IFN-γ | 4.35(3.03-6.72) | 2.69(2.02-3.81) | <0.001*** |
| TNF-α | 3.72(2.64-6.31) | 2.51(1.76-4.67) | <0.001*** |

Date with median and 25th and 75th percentiles

T, T lymphocyte; B, B lymphocyte; NK, Natural killer cell; Th1, T-helper 1 cells; Th2, T-helper 2 cells; Th17, T-helper17 cells; Treg, Regulatory T cells; IL-2, Interleukin-2; IL-4, Interleukin-4; IL-6, Interleukin-6; IL-10, Interleukin-10; IL-17, Interleukin-17; INF-γ, Interferon-γ; TNF-α, Tumor necrosis factor-α.*P<0.05, **P<0.01, ***P<0.001.
