# Supplementary material for: Development and validation of a nomogram for predicting the risk of obstructive coronary artery disease in rheumatoid arthritis patients based on LDL-C, Th17 cells, and IL-17
Source: Front Immunol. 2024 Dec 17;15:1493182. doi: 10.3389/fimmu.2024.1493182 (PMC11685205; doi:10.3389/fimmu.2024.1493182)
Supplement: Supplementary file 3 [file Table3.docx]

| **Supplementary Table 3.** Multivariate logistic regression analyses for factors associated with the presence of Obstructive CAD in RA patients | | | |
| --- | --- | --- | --- |
|  | OR | 95%CI | *P* |
| LDL-C | 11.28 | 3.59-75.97 | 0.003** |
| IL-17 | 1.28 | 1.07-1.63 | 0.006** |
| IgA | 1.36 | 0.79-2.52 | 0.285 |
| IL-4 | 0.99 | 0.97-1.00 | 0.816 |
| Th17 | 1.28 | 1.09-1.60 | 0.003** |
| CRP | 1.02 | 0.99-1.06 | 0.267 |
| IL-2 | 1.18 | 0.53-2.15 | 0.652 |
| IFN-γ | 0.81 | 0.40-1.22 | 0.496 |

OR, Odds ratio; 95%CI, 95% Confidence interval; LDL-C, Low-density lipoprotein cholesterol; IL-17, Interleukin-17; IgA, Immunoglobulin A; IL-4, Interleukin-4; Th17, T-helper17 cells; CRP, C-reactive protein; IL-2, Interleukin-2; INF-γ, Interferon-γ; **p < 0.01.
